# Economic evaluation of supplementing the diet with Souvenaid in patients with prodromal Alzheimer’s disease

**DOI:** 10.1186/s13195-020-00737-9

**Published:** 2020-12-11

**Authors:** Javier Mar, Ania Gorostiza, Oliver Ibarrondo, Igor Larrañaga, Arantzazu Arrospide, Pablo Martinez-Lage, Myriam Soto-Gordoa

**Affiliations:** 1 Basque Health Service (Osakidetza), Debagoiena Integrated Healthcare Organisation, Research Unit, Arrasate-Mondragón, Guipúzcoa Spain; 2 Kronikgune Institute for Health Service Research, Barakaldo, Spain; 3 Biodonostia Health Research Institute, Donostia-San Sebastián, Guipúzcoa Spain; 4 Health Services Research on Chronic Patients Network (REDISSEC), Bilbao, Vizcaya Spain; 5 Unidad de Gestión Sanitaria, Hospital ‘Alto Deba’, Avenida Navarra 16, 20500 Mondragón, Spain; 6 Fundación CITA-Alzheimer Fundazioa, Donostia-San Sebastián, Guipúzcoa Spain; 7 Mondragon Unibertsitatea, Faculty of Engineering, Electronics and Computing Department, Mondragon, Gipuzkoa Spain

**Keywords:** Prodromal Alzheimer’s disease, Souvenaid, Cost-utility, Dementia, Clinical dementia rating

## Abstract

**Background:**

The LipiDiDiet trial showed that Souvenaid, a medical food, might delay progression to dementia in prodromal Alzheimer’s disease (AD). The objective of this study was to assess the cost-utility of Souvenaid compared to placebo in patients with prodromal AD under the conditions applied in that trial.

**Methods:**

A discrete event simulation model was developed based on the LipiDiDiet trial and a literature review to assess the cost-utility of Souvenaid from a societal perspective considering direct and indirect costs. For both intervention and control groups, patient trajectories in terms of functional decline on the Clinical Dementia Rating Sum of Boxes (CDR-SB) scale in LipiDiDiet were reproduced statistically with mixed models by assigning time until events to simulated patients. From the societal perspective, four scenarios were analysed by combining different options for treatment duration and diagnostic test cost. Univariate sensitivity analysis assessed parameter uncertainties.

**Results:**

Validation results at year 2 of disease progression fit with CDR-SB progression in LipiDiDiet. The incremental cost-utility ratio (ICUR) in the baseline case was €22,743/quality-adjusted life year (QALY). All scenarios rendered an ICUR lower than €25,000/QALY (the societal threshold). Moreover, the treatment option was cost-saving and increased health benefits when diagnostic costs were not considered and treatment was only administered during the prodromal stage.

**Conclusions:**

Treating prodromal AD with Souvenaid is a cost-effective intervention in all scenarios analysed. The LipiDiDiet trial showed a modest improvement in disease course but as the social costs of AD are very high, the intervention was efficient. Assessing small benefits at specific stages of AD is relevant because it is reasonable to expect that no effective, safe and affordable disease-modifying therapies will become available in the short to medium term.

## Introduction

Alzheimer’s disease (AD) is a slowly progressing neurodegenerative disease that leads to severe functional disability, the pathological process of AD beginning long before the onset of clinical dementia [1, 2]. Early symptoms such as the loss of episodic memory appear during the mild cognitive impairment (MCI) stage and are associated with synaptic abnormalities [3, 4]. Targeting synaptic dysfunction offers a potential approach to modify the inexorable progression of AD. Data showing that the availability of specific nutrients influences the structure and functionality of neuronal membranes provided a scientific basis to investigate nutritional interventions to delay AD progression [5]. Nonetheless, though the formation of neuronal membrane components in patients with prodromal AD indicates a need to improve their nutritional status, studies have shown that those needs are not currently being covered [6, 7].

Souvenaid® is a medical food for oral consumption under medical supervision with the purpose of addressing disease-specific nutrient requirements. It contains the multinutrient combination Fortasyn™ Connect, which includes precursors and cofactors necessary for forming neuronal membranes that hypothetically serve to support the synthesis of new synapses and maintain existing ones [8]. The LipiDiDiet clinical trial assessed the effectiveness of Souvenaid in prodromal AD, defined according to the International Working Group (IWG)-1 criteria [9, 10]. While no significant effect was observed on performance in the Neuropsychological Test Battery at 2 years, patients who received Souvenaid had significantly better outcomes as measured by the Clinical Dementia Rating Sum of Boxes (CDR-SB) score as well as lower rates of atrophy in magnetic resonance imaging (MRI) [9]. Since the CDR-SB is a reliable and valid staging measure for AD dementia based on cognitive and functional performance [11], these data suggest that treatment with Souvenaid might delay dementia onset in these patients. This is a relevant outcome even when survival is not modified as it would significantly reduce the burden of the disease at the population level [12].

Measuring the cost-utility of treatments evaluated in clinical trials is challenging given their short follow-up. To solve this limitation, simulation models representing the natural history of a disease have proven to be useful tools for analysing future scenarios based on the introduction of new interventions [1, 12]. Medium- and long-term results are determined by the impact of the treatment on key events in the natural history of the disease and by their interaction with other competitive events, known as competitive risks. In the case of AD, the former would be related to the onset of certain stages of the disease, such as MCI or mild dementia, and the latter to non-AD related causes of mortality [1]. As mortality from other causes is not modified, deferring the onset of dementia would lead to patients remaining longer in the less severe stages, increasing patient quality of life and reducing family stress and social costs [12].

Though the LipiDiDiet trial is still ongoing, its 2-year results can be used to carry out an economic evaluation of Souvenaid. In this context, the objective of this study was to assess the cost-utility of Souvenaid compared to placebo in patients with prodromal AD under the conditions applied in the LipiDiDiet trial (International Working Group [IWG]-1 criteria).

## Methods

### Design

A simulation model was developed to represent the natural history of AD in order to assess the cost-utility of Souvenaid. It was programmed in Excel using Visual Basic for Applications syntax. The model compared two cloned cohorts that had differences in progression to dementia according to whether they received Souvenaid (cohort_a_) or not (cohort_b_). It calculated costs in euro (€) and utility in quality-adjusted life years (QALYs) for each patient in both cohorts. The incremental cost, the difference in costs between cohorts, was then calculated as the mean cost in the cohort_a_ (C_a_) minus the mean cost in cohort_b_ (C_b_). Similarly, incremental utility was calculated by subtracting the utility in cohort_b_ (U_b_) from that in cohort_a_ (U_a_). Finally, the incremental cost-utility ratio (ICUR) was estimated by dividing the incremental cost (C_a_-C_b_) by the incremental utility (U_a_-U_b_). The time horizon was the patient’s lifetime, that is, patients were followed until death. The model sums the QALYs and the costs (in euro) incurred by each patient over the entire time horizon of the study. As long-term health benefits and costs are worth less, a discount is applied in economic evaluations to reduce their value annually by a percentage. In our case, a 3% discount rate was applied to costs and QALYs. Since the main economic impact of dementia is related to the care required as patients become dependent, a societal perspective was adopted. This implies incorporating all the costs that society incurs in the care of patients with AD [13]. Data for model parameterization was mainly obtained from the LipiDiDiet trial [9, 14], though some parameters to build the model were taken from the literature.

### The conceptual model

**Fig. 1:**
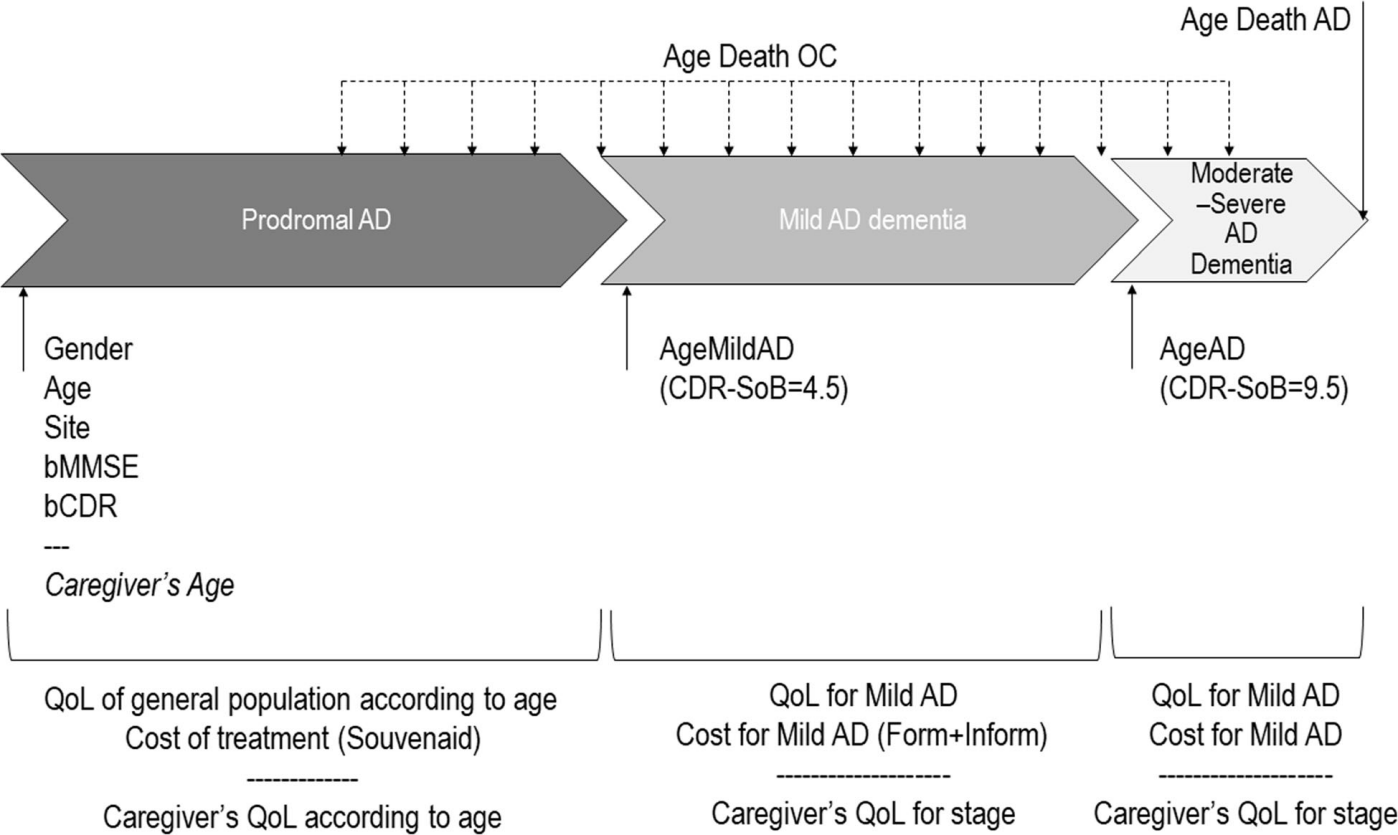
Conceptual model for the cost-effectiveness analysis of Souvenaid in a prodromal AD population. OC, other causes; AD, Alzheimer’s disease; MCI, mild cognitive impairment; QoL, quality of life. Form, formal; Inform, informal; MMSE, Mini-Mental State Examination; CDR-SB, Clinical Dementia Rating Sum of Boxes

In the LipiDiDiet trial [9], only patients with amnestic MCI were eligible. In line with this, in our model, we included a group of patients at the prodromal stage of AD that had the same sex, age, and baseline Mini-Mental State Examination (MMSE) and CDR-SB scores as the trial population described in Soininen et al. [9]. As the disease progressed, these patients might then experience the onset of mild dementia (CDR-SB cut-off of 4.5), then moderate dementia (CDR-SB cut-off of 9.5) [15, 16], die from the disease or, at any time, die from other causes (Fig. 1). The prodromal stage was not associated with any excess risk of death, but when patients reached the dementia stage, they were then considered to have an excess risk of death [17].

### Intervention

Participants in the active group were given the medical food Souvenaid, at a dose of 125 mL once-a-day while they were in the prodromal phase. The control group of the LipiDiDiet trial which was given a placebo was used as a comparator. To maintain motivation, check adherence and monitor safety, participants were contacted by phone throughout the trial (once a month during the first 6 months and every 2 months thereafter).

### Measurement of effectiveness

Based on the results of the LipiDiDiet trial, patient trajectories in terms of functional decline as reflected in CDR-SB scores were reproduced statistically for both intervention and control groups. Following the classification of Bryant et al. [15, 16], the time until dementia was calculated as a function of the progression of cognitive decline (Table 1). As the CDR-SB score worsens more in the control group than the intervention group, it was possible to estimate different conversion rates taking into account this classification [15, 16].

**Table 1:** Model characteristics, population characteristics, costs and utilities

			Source
**Model characteristics**			
Number of patients		100,000	
Number of replications		1	
Discount rate		3%	
Cut-off for mild AD		4.5	[15]
Cut-off for moderate AD		9.5	[15]
**Population characteristics**			
% male		0.46	[9]
Age	Mean (SD)	70.7 (6.2)	[9]
Age_Caregiver_	Mean (SD)	55.2 (14.5)	[18]
Site (%)	1, 2, 3, 4, 5	10, 10, 50, 10, 20	[14]
Baseline MMSE score	Mean (SD)	27.0 (5.3)	[9]
**Annual costs** (**mean**)			[18]
Souvenaid		€1200	
Diagnosis		€2900	[19]
Mild AD (health care)		€3388	
Mild AD (social care)		€13,927	
Mild AD (indirect)		€647	
Mild AD (total)		€17,962	
Moderate/severe AD (health care)		€4659	
Moderate/severe AD (social care)		€34,893	
Moderate/severe AD (indirect)		€820	
Moderate/severe AD (total)		€40,372	
**Utilities**			
MCI	Age 40–49	0.84	[20]
	Age 50–59	0.91	[20]
	Age 60–69	0.90	[20]
	Age 70–79	0.84	[20]
	Age 80–89	0.72	[20]
	Age ≥ 90	0.58	[20]
Mild AD	Mean	0.52	[18]
Moderate AD	Mean	0.21	[18]
MCI caregiver	Mean	Depending on age	[20]
Mild AD caregiver	Mean	0.71	[18]
Moderate AD caregiver	Mean	0.65	[18]

*AD* Alzheimer’s disease, *OC* other causes, *MCI* mild cognitive impairment, *CDR-SB* Clinical Dementia Rating-Sum of Boxes, *MMSE* Mini-Mental State Exam

The health-related quality of life (HRQL) score or utility differed by AD stage (prodromal, mild dementia, moderate dementia and severe dementia) [21]. At the prodromal stage, we assumed the HRQL of this population was the same as that of the general population adjusted for age and sex as shown in Arrospide et al. [20]. At more severe stages, however, cognitive and functional disability decreases HRQL, and to reflect this, data were taken from the Spanish literature [18]. For each patient, the utility-adjusted life expectancy was calculated in QALYs by weighting the time in each stage by its specific utility and the corresponding discount.

### Measurement of costs

We took a societal perspective and considered direct and indirect costs. For this, we used data from Lopez-Bastida et al. [18] who adopted a prevalence-based approach and included healthcare resource use, direct non-healthcare (social care costs) and indirect costs. Total annual costs, updated to 2019, amounted to €17,962 for mild AD and €40,372 for moderate-to-severe AD as shown in Table 1. The costs of the intervention included the diagnostic procedure and medical food which amount to €2900 and €1200 per year, respectively. The diagnostic procedure included the primary care pathway (clinical examination, laboratory tests, computed tomography scan and the MMSE), specialist clinical examination, magnetic resonance imaging (MRI), cerebrospinal fluid tests and neuropsychological examination [19]. As with the calculation of the health benefits, the costs incurred by each patient were calculated by summing the time in each stage weighted by the specific cost and the corresponding discount.

### Parameters

Parameters used to populate the model are listed in Table 1 (basic parameters) and SM1 (basic and statistical parameters, available in the Supplementary Material). Characteristics of the amnestic MCI cohort analysed (*n* = 100,000) were defined according to the features of the LipiDiDiet control group [9]. The attributes considered were age, sex, and baseline MMSE and CDR-SB scores. MMSE and CDR-SB scores were assumed to be correlated taking into account data in the literature [22]. This control group cohort was cloned to generate two populations with identical baseline characteristics. Times until events assigned to the simulated patients, also called entities in the simulation jargon, are dependent on their baseline characteristics. The events considered were death from other causes, onset of mild and moderate-to-severe dementia as defined by CDR-SB scores reaching the corresponding cut-offs and death from dementia. The only group-related parameter was the CDR-SB progression rate.

CDR-SB progression was modelled using the mixed model developed by van Oudenhoven et al. [14]. Mixed models are a variant of regression models that include not only fixed effects but also patient-specific random effects. In this way, they seek to reproduce patient-specific longitudinal profiles by taking into account the fact that repeated measurements from the same patient are more likely to be correlated than measurements from different patients. In our case, the model considers CDR-SB longitudinal data in order to extrapolate the CDR-SB progression results to the longer term. To make the computation more efficient, we did not simulate the monthly decline as done in state-transition models. Rather, we took a discrete event simulation approach in which we defined the time until the event happens, thereby avoiding repeated calculations cycle after cycle. For that reason, we converted the expression of van Oudenhoven et al. [14] as follows:

$$ tUntilEvent=\frac{CDR\  Cut\  Off-{bCDR}_i-{\beta}_0-{\beta}_2{fortasyn}_i-{\beta}_4{mmse}_i-{\beta}_5{site}_i-{b}_{i0}}{\beta_1+{\beta}_3\bullet {fortasyn}_i+{b}_{i1}} $$

where 4.5 and 9.5 are the CDR-SB score cut-offs for mild and moderate AD respectively. All the parameters of the mixed model (β_0_ to β_5_) are described in Table SM1. Each β is the coefficient representing the weight of each of the patient’s characteristics in the CDR-SB progression function.

- bCDR_i_ is the baseline CDR-SB score for patient i
- fortasyn_i_ indicates whether patient i received the treatment or not (0 = no, 1 = yes)
- mmse_i_ is the MMSE score of patient i
- site_i_ is the site at which patient i was treated.

Time from the onset of moderate-to-severe dementia to death was parameterized using data from Dodge et al. [17] (Table SM1). Time until death from other causes was assigned using a specific Gompertz function for each sex [1, 12], namely:

$$ \frac{1}{\beta}\mathit{\ln}\Big(1-\frac{\beta }{\alpha}\ln \left(1-u\right)\bullet {e}^{-\beta \bullet Age} $$

where *α* = *e*^−9,579^for males and *α* = *e*^−10.176^for females and *β* = 0.087 for males and *β* = 0.084 for females.

### Validation

Validation can be defined as a set of methods for judging a model’s accuracy in making predictions [23]. Our model was validated by comparing the modelled disease progression at 2 years and mean change in CDR-SB score at 6, 12, 18 and 24 months as observed by Soininen et al. [9].

### Sensitivity analysis

The model is subject to scenario and parameter uncertainties. In order to address these issues, we carried out a double sensitivity analysis. The scenario sensitivity analysis covers issues related to (1) when treatment is indicated and (2) whether costs of diagnosis are included. First, treatment indication is related to the duration of treatment. So far, in practice, Souvenaid is used until the appearance of mild dementia but it might be also useful during the mild dementia stage. Therefore, we considered two options, namely, stopping the treatment at the onset of mild dementia or at the onset of moderate dementia. Second, diagnostic costs could be deemed to be part of the standard clinical practice (and therefore not included in our cost calculations) or only be required in cases in which treatment is given (and therefore included). Combinations of the aforementioned two factors yielded four scenarios for which the ICURs were estimated. The base case scenario was characterised by a societal perspective, with Souvenaid only administered during the MCI stage, and included diagnostic costs. The societal perspective taken included healthcare costs, informal costs, indirect costs and caregiver quality of life.

The sensitivity analysis estimates the changes in the results related to the parameter uncertainty. For this purpose, a univariate sensitivity analysis was carried out by modifying the base case scenario under different parametric situations as shown in Table 2. The results were presented using a tornado plot which illustrates the impact of each parameter change as the difference that it has on the ICUR calculation compared with the base case.

**Table 2:** Parameters modified in the univariate sensitivity analysis

	Min	Base case	Max	Unit
Annual Souvenaid cost	1000	1200	1400	€/year
Cut-off for mild AD	4	4.5	5	CDR-SB
Cost of mild AD	19,758	14,956	16,166	€/year
Cost of moderate AD	44,409	40,372	36,335	€/year
Cut-off for moderate AD	9	9.5	10	CDR-SB
Moderate AD quality of life	0.23	0.21	0.19	Utility
Mild AD quality of life	0.57	0.52	0.47	Utility
Test cost	2700	2900	3100	€

*AD* Alzheimer’s disease, *CDR-SB* Clinical Dementia Rating Sum of Boxes

## Results

### Validation results

**Fig. 2:**
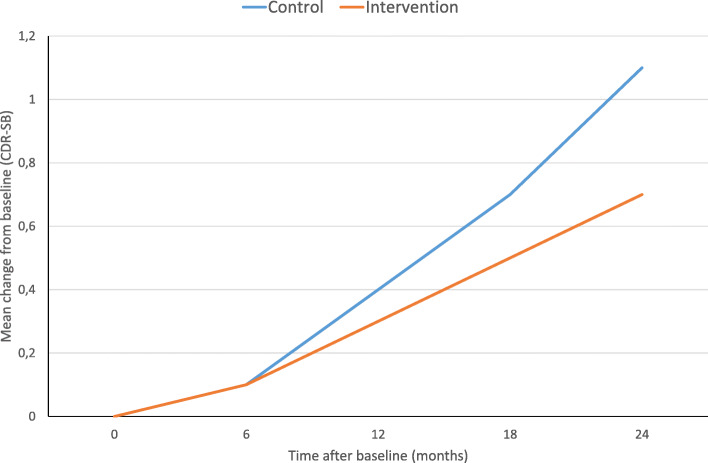
Comparison of mean change in CDR-SB score at 6, 12, 18 and 24 months from the simulation model with that observed by Soininen et al.

Validation results for disease progression after 2 years are presented in Table 3. Figure 2 and Table SM2 (available in the Supplementary Material) show the mean change in CDR-SB score at 6, 12, 18 and 24 months compared with Soininen’s results. Considering the real characteristics of the sample in the simulation model, the rate of progression to dementia at year 2 was 41% in controls and 40% in the intervention group. As the baseline MMSE score was higher in the control group, the results of the two scenarios analysed (same and different baseline) are consistent. When both groups had the same baseline characteristics in the model, the progression rate decreased to 34% in the intervention group, with a mean survival of 8.05 years and more of this time spent with dementia (4.74 years) than with MCI (3.31 years). In the control group, the mean survival was shorter (7.77 years), again this time being distributed between dementia (4.98 years) and MCI (2.79 years) stages. Considering these results, we believe the mixed models reproduce progression accurately and that our model is valid to represent the natural history of patients with prodromal AD.

**Table 3:** Validation results of the dementia progression rate at year 2

% Dementia	Control (%)	Intervention
Results from the LipiDiDiet trial	37	41
Results from the model (real characteristics in control and intervention groups)	41	40
Results from the model (both groups with the same baseline characteristics)	41	36

*CDR-SB* Clinical Dementia Rating Sum of Boxes

Patients with amnestic mild cognitive impairment did not have the same CDR-SB baseline characteristics in the LipiDiDiet trial. In order to overcome this limitation, we adapted intervention group characteristics to the real ones

### Cost-utility results

Table 4 reports the results of the cost-effectiveness analyses under different scenarios. The incremental cost due to Souvenaid was negative in the scenario in which treatment with Souvenaid was only maintained during the MCI stage and which did not consider diagnostic costs but did include informal costs and caregiver quality of life. All the other scenarios produced an ICUR below €25,000/QALY, the willingness-to-pay threshold accepted by the Spanish Health System [24].

**Table 4:** Cost and effectiveness of using Souvenaid under different scenarios

Scenario	Perspective	Treatment indication	Diagnostic test costs	∆ Costs (€)	∆ Effects (QALY)	ICUR (€/QALY)
Baseline	Societal	Only MCI	Included	2633	0.12	22,743
1	Societal	Only MCI	Not included	− 394	0.12	Dominant
2	Societal	MCI and mild AD	Included	7803	0.32	24,392
3	Societal	MCI and mild AD	Not included	4296	0.36	12,076

Societal perspective: includes formal costs, informal costs and caregiver quality of life

*QALY* quality-adjusted life year, *ICUR* incremental cost-utility ratio, *MCI* mild cognitive impairment, *AD* Alzheimer’s disease

**Fig. 3:**
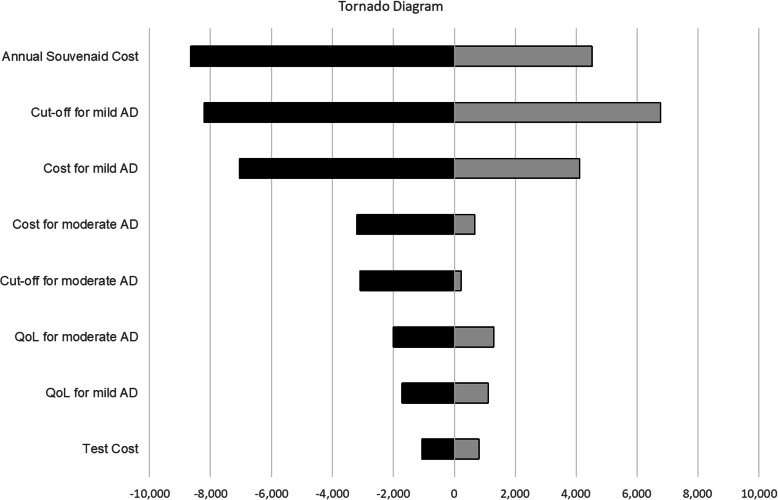
Change in the incremental cost-utility ratio in euros in the univariate sensitivity analysis. AD, Alzheimer’s disease

The tornado diagram in Fig. 3 indicates that the parameters that have the greatest impact on the treatment cost-utility are annual Souvenaid cost, CRD-SB score cut-off for mild AD and mild AD social costs.

## Discussion

The main finding of this economic evaluation is that treating patients with prodromal AD by supplementing their diet with Souvenaid is cost-effective compared to placebo in the scenarios analysed. In the base case scenario, which was characterised by a societal perspective and Souvenaid only being administered during the MCI stage and included diagnostic costs, the treatment can be considered cost-effective in that it has an ICUR of €22,743/QALY, well below the willingness-to-pay threshold in Spain (€25,000/QALY) [24] and in the USA ($100,000/QALY) [25]. This figure represents the cost of generating a QALY within a national health system and provides an estimate of the average opportunity costs of funding decisions.

Analysis of other scenarios showed that the results were sensitive to the inclusion of diagnostic costs and treatment duration. If diagnostic costs were not included, Souvenaid was a dominant treatment. The concept of dominance means that the intervention yields more health measured in QALYs and has lower costs than the control and, according to the decision-making rules, the intervention should be accepted. Maintaining a longer treatment duration and incorporating diagnostic costs with the same effectiveness lowered the efficiency of the treatment. Therefore, we calculated the efficiency of Souvenaid according to both criteria.

It should be underlined that the claim of efficiency can only be applied to the specific group of patients who meet the IWG-1 criteria for prodromal AD and have clinical profiles comparable to the LipiDiDiet population. These patients present with the typical amnestic pattern type of MCI and show at least one positive biomarker (especially a cerebrospinal fluid [CSF] biomarker). As a limitation of our study, we should point out that the new criteria for prodromal AD based on the National Institute of Aging-Alzheimer Association (NIA-AA) guidelines are stricter than the IWG-1 criteria in that the latter include MRI evidence for medial temporal lobe atrophy [9]. The latest NIA-AA criteria limit the requirement of amyloid positivity to positivity in CSF amyloid or amyloid positron emission tomography biomarkers [26].

To advance in the prevention of AD-related dementia, there is a need to identify subpopulations for which each intervention is effective. Mixing patients with different profiles and aetiologies, as is often the case in “MCI”, can easily dilute an effect and hide potential benefits for specific subgroups. On the other hand, translating clinical trial conditions and end-points into measures that are meaningful to policymakers remains a challenge as the resources required to identify the target populations in clinical trials are not always available in clinical practice [19, 27]. In the field of AD, the lack of a so-called disease-modifying therapy usually strengthens physicians’ stance against making an early aetiologic diagnosis when patients present in the prodromal stage before the onset of dependence and dementia. This is more so when the costs of the whole diagnostic process are weighed against the absence of an efficacious intervention. Our analysis has been based on the assumption that the decision to supplement the diet with Souvenaid can only be taken after a specific aetiologic diagnosis has been made, even though including diagnostic costs drives increases in intervention costs and consistently reduces its efficiency [19, 28]. Nevertheless, according to our results, the intervention with Souvenaid would still be cost-effective if diagnostic costs were included. For a suspected AD diagnosis based on CSF results and MRI, Wimo et al. estimated a process cost of €2900 [19].

So far, economic evaluations of dementia treatments have been limited by the lack of benefits in clinical trials [27, 28]. The LipiDiDiet trial has shown, at 2 years, a modest beneficial effect of Souvenaid on patients’ rate of change in a measure that combines cognition and functionality [9]. Evidence of such a benefit has been strengthened by the recent report of significant reductions in cognitive decline as measured by the Neuropsychological Test Battery 5-item composite score over 36 months [29]. Moreover, according to our results, the intervention is efficient, given that AD social costs are very high [28, 30]. In the field of AD health economics, assessing small benefits is relevant because it is reasonable to expect that no effective, safe and affordable disease-modifying therapies will become available in the short term [27]. Developing a disruptive disease-modifying treatment is the great challenge. But for the time being, a multi-modal approach, tackling AD through a combination of several modestly effective but highly safe and tolerable interventions, seems to be the most realistic option for secondary dementia prevention. Diet supplementation for prodromal AD could be combined with multi-domain lifestyle-based interventions to reduce the risk and delay the onset of dependence and dementia from the MCI condition as the starting point [31].

Modelling AD progression especially in the prodromal (pre-dementia) stage always faces the challenge of reproducing the trajectories of groups of patients which are highly heterogeneous in terms of comorbidities, psycho-social conditions and cognitive reserve, as well as genetic and many other factors. Our model only included the progression according to MMSE and CDR-SB scores. A strength of our approach is that the mathematical functions used to reproduce patient trajectories were derived from the same clinical trial, but we recognise that other models have incorporated more dimensions [32, 33]. Comprehensive models include three dimensions (cognitive, functional and behavioural). Our simulated patients were characterised initially by MMSE and CDR-SB scores and then their trajectory relied only on CDR-SB scores, a variable which has shown, with reasonable accuracy, to be able to discriminate between patients with very early AD dementia and those with MCI [15, 16]. The CDR-SB score is deemed appropriate as a measure of global function and seems appropriate in our context, as the effect potentially attributable to Souvenaid is a delay in the progression of AD, specifically in the onset of early dementia, and the concept of dementia refers mostly to the loss of functional abilities to perform everyday cognition-dependent professional, social or family activities [9]. In our opinion, including more dimensions which did not show changes in the LipiDiDiet trial would likely not add value and would go against the principle of modelling of seeking “simpler but not less”.

Given the high goodness of fit between the simulated and the observed mean changes in CDR-SB during the 24-month follow-up, we consider that the technique applied to reproduce the trajectories is adequate. Further, the rate of conversion to dementia recorded in the model also corresponded to that observed in the clinical trial. To achieve that, we applied it to the different baseline CDR-SB scores in each group in the LipiDiDiet trial. On the other hand, to calculate the ICUR, the model was run in both arms with the same baseline CDR-SB (control group scores), because cost-utility is calculated by applying both options to the same target population [13].

## Limitations

Our work has three previously mentioned limitations. First, the old IWG-1 criteria were used to define MCI in the LipiDiDiet trial. Second, Souvenaid did not improve the performance at the primary endpoint (Neuropsychological Test Battery score) in the first 2 years of follow-up in the LipiDiDiet trial. Third, our model did not include the cognitive or behavioural dimensions in the characterisation of the patients, but focused on reproducing the decline in patients with prodromal AD using the CDR-SB, as this was the scale on which scores were modified by Souvenaid treatment.

## Conclusions

Treating prodromal AD with Souvenaid is a cost-effective intervention in all the scenarios analysed. The LipiDiDiet trial showed only a modest improvement in the course of the disease, but as AD social costs are very high, the intervention was found to be efficient. Assessing small benefits is relevant because a multi-modal approach, tackling AD through a combination of several modestly effective but highly safe and tolerable interventions, seems to be the most realistic option for secondary dementia prevention in the absence of a disruptive disease-modifying therapy.

## Trial status

Not applicable

## Supplementary Information

**Additional file 1: Table S1.** Model parameters. **Table S2.** CDR-SB simulated progression in the intervention and control groups.

## Data Availability

No dataset has been used and all the data used are reported in the article or supplementary material.
