# Supplementary material for: Economic evaluation of supplementing the diet with Souvenaid in patients with prodromal Alzheimer’s disease
Source: Alzheimers Res Ther. 2020 Dec 11;12:166. doi: 10.1186/s13195-020-00737-9 (PMC7731786; doi:10.1186/s13195-020-00737-9)
Supplement: Supplementary file 1 — Additional file 1: Table S1. Model parameters. Table S2. CDR-SB simulated progression in the intervention and control groups. [file 13195_2020_737_MOESM1_ESM.docx]

**Supplementary material.**

Table SM1. Model parameters

| **Model characteristics** | | | | | **Source** |
| --- | --- | --- | --- | --- | --- |
|  | Number of patients |  | | 100,000 |  |
|  | Number of replications |  | | 1 |  |
|  | Discount rate |  | | 3% |  |
|  | Cut-off for mild AD |  | | 4.5 | [15] |
|  | Cut-off for moderate AD |  | | 9.5 | [15] |
| **Population characteristics** | | | | |  |
|  | % male |  | | 0.46 | [9] |
|  | Age | Mean (SD) | | 70.7 (6.2) | [9] |
|  | Age_Caregiver_ | Mean (SD) | | 55.2 (14.5) | [20] |
|  | Site (%) | 1, 2, 3, 4, 5 | | 10, 10, 50, 10, 20 | [14] |
|  | Baseline MMSE score | Mean (SD) | | 27.0 (5.3) | [9] |
| **Time to Death OC** | | | | |  |
|  | Male | Alfa | | 6.9E-05 | Gompertz |
|  |  | Beta | | 0.087 |  |
|  | Female | Alfa | | 3.8E-05 |  |
|  |  | Beta | | 0.084 |  |
| **CDR-SB Evolution (Mixed models)** | | | |  | [14] |
|  | β_0_ | Mean (SD) | | 4.6586 (0.4107) |  |
|  | β_1_ | Mean (SD) | | 0.609 (0.047) |  |
|  | β_2_ | Mean (SD) | | 0.071 (0.077) |  |
|  | β_3_ | Mean (SD) | | -0.233 (0.069) |  |
|  | β_4_ | Mean (SD) | | -0.122 (0.015) |  |
|  | β_51_ | Mean (SD) | | 0.6846 (0.0878) |  |
|  | β_52_ | Mean (SD) | | 0.6069 (0.0947) |  |
|  | β_53_ | Mean (SD) | | -0.4693 (0.0958) |  |
|  | β_54_ | Mean (SD) | | 1.7528 (0.1037) |  |
|  | β5_5_ | Mean (SD) | | 0.266 (0.1025) |  |
| **Survival AD** | |  | |  | [17] |
|  | Male |  | |  |  |
|  | Intercept | Mean (SD) | | -87.380 (32.716) |  |
|  | Age | Mean (SD) | | 3.742 (1.156) |  |
|  | Age^2^ | Mean (SD) | | -0.048 (0.014) |  |
|  | Age^3^ | Mean (SD) | | 0.00 (0.00) |  |
|  | Female |  | |  |  |
|  | Intercept | Mean (SD) | | 111.800 (37.957) |  |
|  | Age | Mean (SD) | | -2.560 (1.342) |  |
|  | Age^2^ | Mean (SD) | | 0.019 (0.016) |  |
|  | Age^3^ | Mean (SD) | | -4.25E-05 (6.08E-05) |  |
| **Annual Costs** (**Mean**) | |  | |  | [20] |
|  | Souvenaid |  | | €1,200 |  |
|  | Diagnosis |  | | €2,900 | [21] |
|  | Mild AD (Health care) | | | €3,388 |  |
|  | Mild AD (Social care) | | | €13,927 |  |
|  | Mild AD (Indirect) | | | €647 |  |
|  | Mild AD (Total) | | | €17,962 |  |
|  | Moderate/Severe AD (Health care) | | | €4,659 |  |
|  | Moderate/Severe AD (Social care) | | | €34,893 |  |
|  | Moderate/Severe AD (Indirect) | | | €820 |  |
|  | Moderate/Severe AD (Total) | | | €40,372 |  |
| **Utilities** | |  | |  |  |
|  | MCI | Age 40-49 | 0.84 | | [19] |
|  |  | Age 50-59 | 0.91 | | [19] |
|  |  | Age 60-69 | 0.90 | | [19] |
|  |  | Age 70-79 | 0.84 | | [19] |
|  |  | Age 80-89 | 0.72 | | [19] |
|  |  | Age ≥90 | 0.58 | | [19] |
|  | Mild AD | Mean | | 0.52 | [20] |
|  | Moderate AD | Mean | | 0.21 | [20] |
|  | MCI caregiver | Mean | | Depending on age | [19] |
|  | Mild AD caregiver | Mean | | 0.71 | [20] |
|  | Moderate AD caregiver | Mean | | 0.65 | [20] |

AD: Alzheimer’s Disease; OC: other causes; MCI: mild cognitive impairment; CDR-SB: Clinical Dementia Rating-Sum of Boxes; MMSE: Mini-Mental State Exam.

Table SM2. CDR-SB simulated progression in the intervention and control groups

| Month | CDR-SB Progression | |
| --- | --- | --- |
|  | Control | Intervention CDR_c |
| 0 | 0.0 | 0.0 |
| 6 | 0.1 | 0.1 |
| 12 | 0.4 | 0.3 |
| 18 | 0.7 | 0.5 |
| 24 | 1.1 | 0.7 |
| 30 | 1.4 | 0.8 |
| 36 | 1.7 | 1.0 |

CDR-c: Same baseline CDR-SB scores in both groups.
